# *Viscum album *L. extracts in breast and gynaecological cancers: a systematic review of clinical and preclinical research

**DOI:** 10.1186/1756-9966-28-79

**Published:** 2009-06-11

**Authors:** Gunver S Kienle, Anja Glockmann, Michael Schink, Helmut Kiene

**Affiliations:** 1 Institute for Applied Epistemology and Medical Methodology, Zechenweg 6, D-79111 Freiburg, Germany; 2 Verein Filderklinik e.V, Research Department, Im Haberschlai 7, D-70794 Filderstadt, Germany

## Abstract

**Background:**

*Viscum album *L. extracts (VAE, European mistletoe) are a widely used medicinal plant extract in gynaecological and breast-cancer treatment.

**Methods:**

Systematic review to evaluate clinical studies and preclinical research on the therapeutic effectiveness and biological effects of VAE on gynaecological and breast cancer. Search of databases, reference lists and expert consultations. Criteria-based assessment of methodological study quality.

**Results:**

19 randomized (RCT), 16 non-randomized (non-RCT) controlled studies, and 11 single-arm cohort studies were identified that investigated VAE treatment of breast or gynaecological cancer. They included 2420, 6399 and 1130 patients respectively. 8 RCTs and 8 non-RCTs were embedded in the same large epidemiological cohort study. 9 RCTs and 13 non-RCTs assessed survival; 12 reported a statistically significant benefit, the others either a trend or no difference. 3 RCTs and 6 non-RCTs assessed tumour behaviour (remission or time to relapse); 3 reported statistically significant benefit, the others either a trend, no difference or mixed results. Quality of life (QoL) and tolerability of chemotherapy, radiotherapy or surgery was assessed in 15 RCTs and 9 non-RCTs. 21 reported a statistically significant positive result, the others either a trend, no difference, or mixed results. Methodological quality of the studies differed substantially; some had major limitations, especially RCTs on survival and tumour behaviour had very small sample sizes. Some recent studies, however, especially on QoL were reasonably well conducted. Single-arm cohort studies investigated tumour behaviour, QoL, pharmacokinetics and safety of VAE. Tumour remission was observed after high dosage and local application. VAE application was well tolerated. 34 animal experiments investigated VAE and isolated or recombinant compounds in various breast and gynaecological cancer models in mice and rats. VAE showed increase of survival and tumour remission especially in mice, while application in rats as well as application of VAE compounds had mixed results. *In vitro *VAE and its compounds have strong cytotoxic effects on cancer cells.

**Conclusion:**

VAE shows some positive effects in breast and gynaecological cancer. More research into clinical efficacy is warranted.

## Background

Breast and gynaecological cancers (i.e. ovarian, endometrial, cervical, vaginal, vulval, and fallopian cancers) account for a significant amount of morbidity and mortality in women. In Europe an estimated 429,900 cases were diagnosed as breast cancer in 2006 (13.5% of all cancer cases) and 131,900 died from it, despite substantially improved treatment options (surgery, chemotherapy, radiation, hormonal and targeted therapies) [1]. Of female cancer survivors more than half had suffered from breast or gynaecological cancer [2].

40% to 80% of these patients use complementary therapies additionally to well-established treatments [3-8]. This includes a variety of medicinal plants, but also acupuncture, psychosocial support, yoga, art therapies and others. These are supportive measures to control symptoms, improve quality of life, boost the immune system, and possibly prolong life. Sufficient evaluation is often lacking, however, of the extent to which these therapeutic goals are achieved, as well as of issues relating to safety and mode of action. Medicinal plants in particular have a long history in the treatment of cancer and other conditions connected with tumours, and also play a major role in the development of new drugs today. Over 60% of currently used anti-cancer agents originally derive from natural sources such as plants, marine organisms and micro-organisms [9].

Across Europe, *Viscum album *L. extracts (VAE or European mistletoe, not to be confused with the *Phoradendron *species or "American mistletoe") are among the most common herbal extracts applied in cancer treatment [3,7,8,10]. *Viscum album *is a hemi-parasitic shrub and contains a variety of biologically active compounds. Mistletoe lectins (ML I, II and III) have been most thoroughly investigated. MLs consist of two polypeptide chains: a carbohydrate-binding B-chain that can bind on cell surface receptors, which enables the protein to enter the cell [11-13]; and the catalytic A-chain which can subsequently inhibit protein synthesis, due to its ribosome-inactivating properties, by removing an adenine residue from the 28S RNA of the 60S subunit of the ribosome [11]. Other pharmacologically relevant VAE compounds are viscotoxins and other low molecular proteins, VisalbCBA (*Viscum album *chitin-binding agglutinin) [14], oligo- and polysaccharids [15,16], flavonoids [17], vesicles [18], triterpene acids [19], and others [20,21]. Whole VAE as well as several of the compounds are cytotoxic and the MLs in particular have strong apoptosis-inducing effects [22-24]. MLs also display cytotoxic effects on multidrug-resistant cancer cells (e.g. *MDR*+ colon cancer cells [25]) and enhance cytotoxicity of anticancer drugs [26,27]. In mononuclear cells VAE also possess DNA-stabilizing properties. VAE and its compounds stimulate the immune system (*in vivo *and *in vitro *activation of monocytes/macrophages, granulocytes, natural killer (NK) cells, T-cells, dendritic cells, induction of a variety of cytokines such as IL-1, IL-2, IL-4, IL-5, IL-6, IL-8, IL-10, IL-12, GM-CSF, TNF-α, IFN-γ (overview see [20,21]). The cytotoxicity of human natural and lymphokine-activated killer cells, for instance, can be markedly enhanced *in vitro *by VAE rhamnogalacturonans, which bridge these killer cells with NK-sensitive or insensitive tumour cells [28,29]. Furthermore, VAE seem to interfere with tumoural angiogenesis [30,31]. Injected into tumour-bearing animals, VAE and several of their compounds (MLs, a 5 kDa protein not specified further, protein complexes isolated by Vester and colleagues, oligosaccharids) display growth-inhibiting and tumour-reducing effects [20,21]. Despite extensive experimental analyses of their biological properties, many questions regarding the precise mode of action of VAE still remain.

For clinical application VAE are made from mistletoes grown on different host trees [Host trees of VAE: Fir (*Abies*, A); maple (*Acer*, Ac); almond tree (*Amygdalus*, Am); birch (*Betula*, B); whitethorn (*Crataegus*, C); ash tree (*Fraxinus*, F); appletree (*Malus*, M); pine (*Pinus*, P); poplar (*Populus*, Po); oak (*Quercus*, Qu); willow (*Salix*, S); lime (*Tilia*, T), elm (*Ulmus*, U)], either by aqueous extraction, partly combined with fermentation, or by pressing procedures. Depending on host tree, harvesting time and extraction procedure, VAE vary in regard to their active compounds and biological properties. Different commercial VAE preparations are available, and a recombinant ML (rML) drug is currently being developed and tested in clinical trials [32,33].

Clinical effects of VAE in cancer have been investigated in a variety of studies and assessed in systematic reviews [34-39]. These reviews, however, had inconsistent results, they are outdated, incomplete or concentrate on partial aspects. No review has yet assessed clinical and preclinical effects specifically and comprehensively for breast and gynaecological cancer, although there is widespread usage in these patients [3,7]. Our primary aim was therefore to assess the potential therapeutic effectiveness of VAE, and their potential biological effects on breast and gynaecological cancer in clinical and preclinical studies.

## Methods

### Design

Systematic review of clinical and preclinical studies investigating the influence of VAE on breast or gynaecological cancer.

### Search strategy

We used a systematic process to search the following databases for clinical trials – AMED, Biosis Previews, Cochrane Library (Cochrane Database of Systematic Reviews, Cochrane Controlled Trials Register, The NHS Economic Evaluation Database, Health Technology Assessment Database), Embase, Medline/Premedline, NLM Gateway, private databases – from inception of these databases to December 2008 using the terms (MISTLETOE OR VISCUM? OR MISTEL? OR ISCADOR? OR ISCAR OR HELIXOR OR ABNOBA? OR ISCUCIN OR ISOREL OR VISOREL OR ?SOREL OR WELEDA OR WALA OR EURIXOR OR LEKTINOL OR PLENOSOL OR AVISCUMINE) AND (STUDY? OR STUDIE? OR TRIAL OR EVALUAT? OR RANDOM? OR INVESTIG? OR COHORT? OR KOHORT? OR OUTCOME?). The reference list from each potentially eligible study, relevant review article and textbook was checked, and experts in the field and manufacturers of mistletoe preparations were contacted for additional reports.

Regarding *in vitro *or *in vivo *(animal) experiments on anticancer effects, we checked title and abstract, and, where necessary, the whole article of each VAE-related reference in the databases (Medline/Pubmed and comprehensive private databases, using above mentioned terms but without restriction to clinical studies) and in major surveys.

### Selection

The following selection criteria were used for inclusion of studies in the analysis: (I) prospective randomized or non-randomized controlled clinical trial, or prospective single-arm cohort study (e.g. phase II trial) or pharmaco-epidemiological cohort study; (II) study population with breast or gynaecological cancer, i.e. ovary, uterus, cervix, genital cancer, or cervical intraepithelial neoplasm (CIN); (III) intervention group treated with VAE preparation; (IV) clinically relevant outcome (i.e. survival, disease-free interval, remission, relapse, QoL, or reduction of side effects or immune suppression during cytoreductive therapy); (V) completion of study; (VI) published or unpublished. Studies were excluded if they: only measured toxicity or tolerability (phase I trial), only measured stimulation of immunological parameters, were not conducted on cancer patients, or had a retrospective design (except pharmaco-epidemiological cohort studies). There were no restrictions on language.

For *in vitro *and animal experiments the criteria were adapted accordingly; unpublished material was not included however. *In vitro *experiments were restricted to cancer cells originating from human tumours.

### Validity assessment and data abstraction

Criteria-based analysis was performed on the selected clinical studies to assess their methodological quality. Analyses were performed independently by two reviewers (GK, HK). There were no major differences in study assessment; disagreements were resolved by discussion. Criteria for assessing strength of evidence in controlled trials were adapted from the National Health Service Centre for Reviews and Dissemination [40] and from criteria for good methodology as already applied in earlier reviews on VAE trials [34,36,41]. Quality criteria were adjusted for cohort studies [36]. Data were abstracted by one reviewer (GK) and checked by a second reviewer (AG). When necessary, primary authors of the trials were contacted for additional information.

Regarding animal experiments we extracted data on study size, animal model, tumour type, tumour transfer, intervention, treatment schedule, outcome, physiological monitoring, side effects, dose-response, randomization, control treatment, blinding of outcome assessment, publication in a peer-reviewed journal, and funding source.

## Results

### Result of literature search

The literature search identified 306 references describing potential clinical studies (after deletion of duplicates). After deleting references only describing studies on immune modulation or toxicity or tolerability (phase I trial), or only on cancer sites other than breast or gynaecological, with retrospective evaluation, without quantification of results, or only investigating complex treatment regimes, or describing studies already published elsewhere, 48 potential studies were identified that met the inclusion criteria. Two trials [42,43], conducted in Poland, were excluded because of severe validity concerns: a collaborating scientist questioned the alleged randomization of treatment allocation, and no information could be obtained from the authors to clarify this question. One further RCT (on Lektinol^® ^and breast cancer by Schwiersch et al.) might have met the inclusion criteria but was unpublished and unavailable. Thus it was possible to include 46 studies in this review: 19 RCTs, 16 non-RCTs, and 11 single-arm cohort studies. Of the 46 studies, 43 were published (4 of these only as an abstract), 1 study was retrieved as a doctoral dissertation, and 2 were unpublished reports.

1632 VAE-related references were checked by title, abstract or whole article, book chapter, or book regarding *in vitro *or animal studies. Experiments meeting the inclusion criteria were excluded if they were described in another publication, were not published in a scientific journal, scientific book or as a scientific dissertation, were unavailable (some dissertations from the 1950s and 60s), or if they did not present sufficient information.

### Characteristics of included clinical studies

Tables 1, 2, 3, 4, 5, and 6 show characteristics of the clinical studies. Settings of the studies were mostly academic hospitals, large community hospitals, and specialized cancer hospitals. The studies were mainly conducted in Germany, but also in Austria, Switzerland, USA, Serbia, Russia, Bulgaria, Ukraine, Italy, Egypt, Israel, China, South Korea. Most studies were conducted in more than one centre. In 31 of the 32 studies published since 2000, the funding source was identifiable: three studies had public funding [44-46], 17 a combination of public and industry funding, and 11 industry funding alone.

**Table 1:** Randomized Controlled Clinical VAE Trials on Breast and Gynaecological Cancer: Quality Assessment

**Author, Year**	**Quality Criteria Fulfilled in Studies**^I^											**Participants**	**AR**^II^
			
	**A)**	**B)**	**C)**	**D)**	**E)**	**F)**	**G)**	**H)**	**I)**	**J)**	**K)**		
Tröger 2009 [47]	+	-	-	(+)	+	+	+	+	(+)	(+)	+	95	6%
Büssing 2008 [48]	+^III^	-^III^	-^III^	-^III^	(-)^III^	-^III^	(-)^III^	(-)^III^	(-)^III^	(-)^III^	-^III^	65	No data
Grossarth 2008a [49]	+	+	-	(-)	+	(-)	+	(+)	+	+	-	76	21%
Grossarth 2008b [49]	+	+	-	(-)	+	+	+	(+)	+	+	-	52	0%
Grossarth 2007a [50]	+	+	-	(-)	+	(-)	+	(+)	+	+	-	50	16%
Grossarth 2007b [50]	+	+	-	(-)	+	(-)	+	(+)	+	+	-	48	17%
Grossarth 2007c [51]	+	+	-	(-)	+	+	+	(+)	+	+	-	38	0%
Grossarth 2006a [52,53]	+	+	-	(-)	+	(-)	+	(+)	+	+	-	118	36%
Semiglasov 2006 [54]	+	-	(+)	(+)	+	+	+	+	+	+	+	352	4%
Auerbach 2005 [55]	+	-	(+)	(+)	+	-	+	(+)	+	(+)	+	23	30%
Piao 2004 [56]	+	+	-	(-)	+	+	+	(+)	+	+	+	233	4%
Semiglasov 2004 [57]	+	-	(+)	(+)	+	+	+	+	+	+	+	272	4%
Borrelli 2001 [58]	+	-	(+)	(+)	+	+	(+)	+	(-)	(+)	-	30	0%
Grossarth 2001a [59]	+	+	-	(-)	+	+	+	(-)	+	+	-	34	0%
Grossarth 2001b [59]	+	+	-	(-)	+	(-)	+	(-)	+	+	-	98	20%
Kim 1999 [60]	+	-	-	-	(-)	-	(+)	(+)	(-)	(-)	-	30^IV^	13%
Heiny 1991 [61]	+	-	(-)	(-)	+	(+)	+	(+)	+	+	-	46	13%
Gutsch 1988 [62]	+	-	-	(-)	+	(-)	+	+	(+)	+	-	692	20%
Lange 1985 [63]	+	+	-	(-)	+	(-)	+	(+)	+	+	-	68	35%

^I ^A) Protection against selection bias, especially by adequate randomization

B) Minimization of heterogeneity by pre-stratification or matching

C) Protection against observer bias by blinding of patient, care provider, and outcome assessor

D) Protection against performance (treatment) bias by standardization of care protocol, documentation of all co-interventions, blinding of patients and care providers

E) Protection against measurement (detection) bias by standardization of outcome assessment

F) Protection against attrition (exclusion) bias, lost patients <10% or by intention-to-treat analysis (including non-adherers as randomized) plus per-protocol analysis (excluding non-adherers) in combination with sensitivity analysis, and by comparison of prognostic characteristics of lost patients and compliers

G) Effect measurement relevant and well described

H) Well-described intervention, patient characteristics, disease (diagnosis, stage, duration), previous therapy

I) Well-described study design

J) Well-described results

K) Data quality assured by ICH-GCP guidelines, especially by monitoring

+ = adequately fulfilled, (+) = partly fulfilled, (-) = little fulfilled, - = not fulfilled

^II ^AR: attrition rate (dropouts, protocol deviations, withdrawals, patients did not receive treatment as allocated).

^III ^Assessment based only on an abstract

^IV ^Discrepancy in patient numbers in two presentations (30 and 33), with corresponding discrepancy of results

**Table 2:** Non-Randomized Controlled Clinical VAE Studies on Breast and Gynaecological Cancer: Quality Assessment

**Author, Year**	**Quality Criteria Fulfilled in Studies**^I^											**Participants**	**AR**^II^	**Design/control for confounding**
				
	**A)**	**B)**	**C)**	**D)**	**E)**	**F)**	**G)**	**H)**	**I)**	**J)**	**K)**			
Grossarth 2008c [49]	(+)	+	-	(-)	+	+	+	(+)	+	+	-	200	5%	Prospective pair-matching
Grossarth 2008d [49]	(+)	+	-	(-)	+	(-)	+	(+)	+	+	-	282	27%	Prospective pair-matching
Loewe-Mesch 2008 [64]	-	-	-	(-)	+	(-)	+	+	(+)	+	-	82	20%	Self-selected treatment allocation, no adjustment
Grossarth 2007d [50]	(+)	+	-	(-)	+	(-)	+	(+)	+	+	-	198	24%	Prospective pair-matching
Grossarth 2007e [50]	(+)	+	-	(-)	+	+	+	(+)	+	+	-	132	6%	Prospective pair-matching
Grossarth 2007f [51]	(+)	+	-	(-)	+	+	+	(+)	+	+	-	212	4%	Prospective pair-matching
Grossarth 2007g [51]	(+)	+	-	(-)	+	+	+	(+)	+	+	-	140	6%	Prospective pair-matching
Grossarth 2006b [52,53]	(+)	+	-	(-)	+	(-)	+	(+)	+	+	-	210	20%	Prospective pair-matching
Büssing 2005 [65]	(-)	-	-	(-)	+	+	(+)	(+)	(+)	+	+	105	7%	Comparison of two different hospitals. Pair-matching for analysis
Grossarth 2001c [59]	(+)	+	-	(-)	+	+	+	-	+	+	-	792	4%	Prospective pair-matching
Salzer 1987 [66]	(+)	-	-	(-)	+	-	+	-	-	(+)	-	155	not shown	Alternating treatment allocation
Fellmer 1966 [67]	-	-	-	(-)	+	-	+	+	-	-	-	924	15%	Treatment allocation by neutral attending physician
Majewski 1963 [68]	(+)	-	-	(-)	+	-	+	-	-	-	-	^III^	not shown (15%)^IV^	Alternating treatment allocation

Retrolective pharmaco-epidemiological cohort studies														

Beuth 2008 [69]	-	(+)	-	-	(-)	-	(+)	(-)	(+)	(+)	+	681	^V^	Multivariate adjustment only for one main outcome ("complaints")
Bock 2004 [70]	-	(+)	-	-	(-)	-	(+)	+	(+)	(+)	+	1442	^V^	Multivariate adjustment
Schumacher 2003 [71,72]	-	(+)	-	-	(-)	-	(+)	+	(+)	(+)	+	689	^V^	Propensity score adjustment

^I–II ^Abbreviations as in Table 1.

^III ^Number of study patients not indicated; mistletoe group included 155 patients.

^IV ^Numbers given only for mistletoe group.

^V ^Not applicable for retrolective studies.

**Table 3:** Controlled Clinical Studies on VAE Treatment in Breast and Gynaecological Cancer: Survival

**Site**	**Stage**	**Intervention (evaluable patients)**	**Survival Outcomes**					**Author, year, reference**
				
			**Years (median)**	**Hazard ratio**	**5-year survival and others**	**P-value**	**95% CI**	
**Randomized controlled trials**								

Breast	T1a-3, N0, M0	Iscador (38)	14.8	0.65		0.2	0.34–1.25	Grossarth 2006a [52,53,135]
							
		None (38)	13.8					

	IIIA–IIIB	Iscador (17)	6.3	0.46		0.13	0.16–1.31	Grossarth 2001a [59,135,166]
							
		None (17)	2.3					

	T1-3, N0-3, M0, local recurrence	Surgery, radiation^I^, Helixor (192)	Not applicable^II^		69.1% 5-year survival	0.048		Gutsch 1988 [62]
							
		Surgery, radiation^I^, CMF (177)			67.7% 5-year survival	0.025		
							
		Surgery, radiation^I ^(274)			59.7% 5-year survival			

Breast, others	All stages	Iscador (39)	3.5 (mean)			0.04		Grossarth 2001b [59]
							
		None (39)	2.5 (mean)					

Cervix	IVA-B	Iscador (19)	1.83	0.46		0.12	0.18–1.21	Grossarth 2007c [51]
							
		None (19)	1.92					

Uterus	IA-C	Iscador (30)	6.29	0.36		0.014	0.16–0.82	Grossarth 2008a [49]
							
		None (30)	5.17					

	IVA-B	Iscador (26)	1.5	1		0.99	0.46–2.16	Grossarth 2008b [49]
							
		None (26)	2.0					

Ovary	IA–IC	Iscador (21)	6.75	0.40		0.058	0.15–1.03	Grossarth 2007a [50]
							
		None (21)	5.58					

	IV	Iscador (20)	2.75	0.33		0.033	0.12–0.92	Grossarth 2007b [50]
							
		None (20)	1.58					

**Non-randomized controlled studies**								

Breast	T1-3, N0, M0	Iscador (84)^III^	11.75	0.42		0.0002	0.27–0.68	Grossarth 2006b [52,53,135]
							
		None (84)	10.13					

	Local recurrence, N0, M0	Iscador (29)^IV^	5.17			0.0025		Grossarth 2001b [59,135]
							
		None (29)	4.33					

	T1-4, N>1, M0	Iscador (38)^IV^	4.04			0.0516		Ø same study
							
		None (38)	3.17					

	TX, NX, M1	Iscador (53)^IV^	3.08			0.0056		Ø same study
							
		None (53)	2.17					

	I–III	Iscador, (76)			29% alive 1985, after 11–14 years	not shown		Salzer 1987 [66]
								
		Radiation, hormone (79)			24% alive 1985, after 11–14 years			

Cervix	IB-IVA	Iscador (102)^III^	7.17	0.41		<0.0001	0.27–0.63	Grossarth 2007f [51]
							
		None (102)	5.92					

	IV	Iscador (66)^III^	2.33	0.54		0.015	0.32–0.89	Grossarth 2007g [51]
							
		None (66)	1.83					

	I–III	Radiation, Iscador (81)			83% 5-year survival	0.05		Fellmer 1966 [67]
								
		Radiation (709)			69% 5-year survival			

Uterus	IIIA–IVB	Iscador (95)^III^	2.75	0.61		0.023	0.39–0.93	Grossarth 2008c [49]
							
		None (95)	1.67					

	IA-C	Iscador (103)^III^	8.75	0.41		<0.0001	0.26–0.63	Grossarth 2008d [49]
							
		None (103)	6.67					

Ovary	IA–IC	Iscador (75)^III^	6.83	0.47		0.0002	0.31–0.69	Grossarth 2007d [50]
							
		None (75)	5.83					

	IV	Iscador (62)^III^	1.79	0.62		0.077	0.37–1.05	Grossarth 2007e [50]
							
		None (62)	1.17					

Genital	All stages	Surgery^I^, radiation^I^, Iscador (155)			Disease-specific survival partly improved	not shown		Majewski 1963 [68]
								
		Surgery^I^, radiation^I^,(not shown)						

**Retrolective pharmaco-epidemiological cohort studies**								

Breast	I–III	Conventional therapy, Iscador (710)		0.46		0.038	0.22–0.96	Bock 2004 [70]
								
		Conventional therapy (732)						

	I–IV	Conventional therapy, Eurixor (219)			No difference observed^V^			Schumacher 2003 [71,72]
								
		Conventional therapy (470)						

^I ^Co-intervention (i.e. radiation) applied to part of the group

^II ^Not applicable since more than 50% alive at study termination

^III ^Data from complete set of patient pairs reported

^IV ^Data only from patient pairs with strict matching reported

^V ^No difference could be found due to limited observation time (median < 10 months)

CMF: Cyclophosphamide, methotrexate, 5-fluorouracil

P-value, 95% CI (confidence interval): Statistical significance of difference between mistletoe (or other verum) and control group.

**Table 4:** Controlled Clinical Studies on VAE Treatment in Breast and Gynaecological Cancer: Tumour Behaviour or Pleurodesis

**Site**	**Stage**	**Intervention (evaluable patients)**	**Outcome**	**P-value**	**95% CI**	**Author, year, reference**
**REMISSION**						

**Randomized controlled trials**						

Breast, ovary, lung	T_1–4_, N_0–3_, M_0–1_	Chemotherapy^I^, Helixor A (115)	Remission rate: no difference			Piao 2004 [56]
						
		Chemotherapy^I^, Lentinan (109)				

Ovary, others	Inoperable	Radiation, cisplatin, holoxan, Helixor (23)	10% complete remission 48% partial remission 5% progress			Lange 1985 [63]
					
		Radiation, cisplatin, holoxan (21)	17% complete remission 48% partial remission 4% progress			

Pleural effusion^II^	Advanced	Helixor (11)	82% complete remission 9% partial remission	<0.05 ^III^		Kim 1999 [60]
					
		Doxycycline, meperidine, lidocaine (15)	40% complete remission 27% partial remission			

**DISEASE-FREE INTERVAL, TIME TO EVENT, RECURRENCE (HAZARD RATIO)**						

**Randomized controlled trials**						

Breast	T_1a-3_, N_0_, M_0_	Iscador (38)	Time to local recurrences: 0.44 lymphatic metastases: 0.27 distant metastases: 0.50 all events (incl.death) 0.65	0.18 0.0048 0.061 0.012	0.14–1.44 0.11–0.67 0.24–1.03 0.47–0.91	Grossarth 2006a [52,53]
						
		None (38)				

**Non-randomized controlled trials**						

Breast	T_1–3_, N_0_, M_0_	Iscador (84)	Time to local recurrences: 0.42 lymphatic metastases: 0.22 distant metastases: 0.36 all event (incl.death) 0.66		0.21–0.83 0.10–0.47 0.21–0.62 0.55–0.79	Grossarth 2006b [52,53]
						
		None (84)				

Cervix	IB-IVA	Iscador (102)	Time to local recurrences: 1.42 lymphatic metastases: None distant metast.:1 in Iscador group all event (incl.death) 0.32	0.61 n.a. n.a. <0.0001	0.37–5.39 n.a. n.a. 0.22–0.48	Grossarth 2007f [51]
						
		None (102)				

**Retrolective pharmaco-epidemiological cohort study**						

Breast	I–III	Conventional therapy, Helixor (167)	Recurrence, metastases, reoperation: no difference			Beuth 2008 [69]
						
		Conventional therapy (514)				

	I–III	Conventional therapy, Iscador (710)	Recurrence: 0.98 Dist. metast. 0.65	0.947 0.172	0.60–1.62 0.35–1.21	Bock 2004 [70]
						
		Conventional therapy (732)				

	I–IV	Conventional therapy, Eurixor (219)	Time to relapse: 0.28	0.012	0.10–0.76	Schumacher 2003 [71,72]
						
		Conventional therapy (470)				

^I ^Chemotherapy: see table 5

^II ^Plural effusion indicates treatment site (primary cancer site: 4 × breast, 1 × cervix, 23 × lung, 1 × stomach, 1 × unknown primary)

^III ^Side effects in Helixor and doxocycline group: pain in 6 and 14, fever in 3 and 6, burning sensation in 0 and 5 patients respectively; difference statistically significant (p < 0.05)

P-value, 95% CI (confidence interval): Statistical significance of difference between mistletoe (or other verum) and control group.

**Table 5:** Controlled Clinical Studies on VAE Treatment in Breast and Gynaecological Cancer: Reduction of side effects of chemotherapy, radiation or surgery; QoL

**Site**	**Stage**	**Intervention (evaluable patients)**	**Reduction of side effects of chemotherapy, radiation or surgery**			QoL (*during chemotherapy, radiation)						**Author, year, reference**
				
			**Outcome**		**P-value**	**Measurement scale and outcome**				**P-value**	**95% CI**	
**Randomized controlled trials**												

Breast	T_1–3_, N_0–2_, M_0_	CAF, Iscador or Helixor (59)	Neutropenia	15%	0.195	EORTC QLQ-C30* (Pain*, diarrhoea*, role*, insomnia*, nausea/vomiting*)				0.0438 to 0.0003		Tröger 2009 [47]
												
		CAF (30)		27%								

	No data	(F)EC, Iscador M (32)	EC-associated inhibition of granulocyte function: no difference. Reduction of EC-related side effects (nausea, constipation, pain, stomatitis). Lymphocytes, retching, emesis: no difference		>0.27	EORTC QLQ-C30*, BR 23*, Rhodes Index*: no difference				No data	No data	Büssing 2008 [48]
												
		(F)EC (33)			"significant"							

	T_1a-3_, N_0_, M_0_	Iscador (38)				Self-regulation questionnaire, Hazard-ratio			0.35		0.05–0.60	Grossarth 2006a [52,53]
												
		None (38)										

	T_1–3_, N_0_-N_+_, M_0_	CMF, Lektinol 15 ng ML (169)	Haematological parameters, hospitalization, paracetamol, metoclopramid: no difference. Leucopenia ↓ (trend)			FACT-G* ↑ 4.4	GLQ-8* sum ↓ 28.9	Spitzer uniscale* ↓ 12.2	KPS* No difference	<0.0001		Semiglasov 2006 [54]
										
		CMF, placebo (168)				FACT-G* ↓ 5.11	GLQ-8* sum ↑ 94.8	Spitzer uniscale* ↑ 10.8				

	T_1–2_, N_0–1_, M_0_	CMF, radiation, Helixor A (11)	CMF-induced NK-cell decrease ↓ SCE-increase ↓ other immune markers: no difference		0.005 n.s.	EORTC QLQ-C30*		No difference, data not shown		not shown		Auerbach 2005 [55]
										
		CMF, radiation, placebo (9)										

	T_1–3_, N_0_-N_+_, M_0_	CMF, Lektinol 5 ng ML (66)	Haematological parameters, hospitalization, paracetamol, metoclopramid: no difference. immune markerers: CD4, CD4/CD8, NK-cell-activity: significant ↑			GLQ-8* sum No difference		Spitzer uniscale* No data	QLQ C-30* No difference	<0.05		Semiglasov 2004 [57]
										
		CMF, Lektinol 15 ng ML (65)				GLQ-8* sum Superior 60,8mm		Spitzer uniscale* Superior 16,4 mm				
										
		CMF, Lektinol 35 ng ML (64)				GLQ-8* sum No difference		Spitzer uniscale* No data				
										
		CMF, placebo (66)										

	IIIA–IIIB	Iscador (17)				Self-regulation questionnaire (score 1–6)		2.92 → 3.7		0.13		Grossarth 2001a [59]
											
		None (17)						2.87 → 2.99				

	IV	Iscador spezial (20)				Spitzer score questionnaire		~5 → 7.2		<0.05		Borrelli 2001 [58]
											
		Placebo (10)						~5.2 → 4.8				

	Advanced	VEC, Eurixor (21)	Leukopenia ↓ Platelets: no difference		≤ 0.001	QoL index* (superior)		Anxienty scale* (superior)		≤ 0.01		Heiny 1991 [61]
												
		VEC, placebo (19)										

Breast, others	All stages	Iscador (39)				Self-regulation questionnaire (score 1–6)		3.41 → 3.87		0.02		Grossarth 2001b [59]
											
		None (39)						3.85 → 3.62				

Breast, ovary, lung	T_1–4_, N_0–3_, M_0–1_	Chemotherapy^I^, Helixor A (115)	Chemotherapy-related adverse events 28		not shown	FLIC-score* ↑ 9	TCM-score* ↑ -1		KPS* increase in % of patients 50%	FLIC 0.014 TCM 0.0007 KPS 0.002		Piao 2004 [56]
							
		Chemotherapy^I^, Lentinan (109)	Chemotherapy-related adverse events 77			FLIC-score* ↑ 4,7	TCM-score* 0		KPS* increase in % of patients 32%			

Ovary	IA–IC	Iscador (21)				Self-regulation questionnaire, (score 1–6) median difference			0.58	0.0002	0.30–0.90	Grossarth 2007a [50]
												
		None (21)										

Ovary, others	Inoperable	Radiation, cisplatin, holoxan, Helixor (23)	Nausea ↓, vomiting ↓, depression of leucopoiesis ↓		0.005, 0.08, 0.003	KPS*	67% → 76% (p = 0.0008^II^)			not shown		Lange 1985 [63]
										
		Radiation, cisplatin, holoxan (21)					70% → 74% (p = 0.12^II^)					

Cervix	IVA-B	Iscador (19)				Self-regulation questionnaire, (score 1–6) median difference		0.7		0.014	0.15–1.05	Grossarth 2007c [51]
												
		None (19)										

Uterus	IA-C	Iscador (30)				Self-regulation questionnaire, (score 1–6) median difference		0.4		0.0012	0.15–0.70	Grossarth 2008a [49]
												
		None (30)										

**Non-randomized controlled studies**												

Breast	T_1–3_, N_0_, M_0_	Iscador (84)				Self-regulation questionnaire Hazard-ratio		0.20		0.031	0.00–0.35	Grossarth 2006b [52,53]
												
		None (84)										

	I–II	Surgery, CMF/EC, Iscador (33)	CMF/EC-induced lymphocyte decrease ↑, platelet decrease ↓		n.s, 0.01	EORTC QLQ-C30*, BR 23*		Reduced increase of nausea/vomiting, general side effects of CMF/EC		0.02 0.02		Loewe-Mesch [64]
												
		Surgery, CMF/EC (33)										

Breast (suspected)		Surgery, Iscador M spezial (47)	Prevention of surgery-associated inhibition of granulocyte function (PMA- and E.coli-stimulated oxidative burst)		<0.0001,<0.001							Büssing 2005 [65]
												
		Surgery (51)										

Ovary	IA–IC	Iscador (75)				Self-regulation questionnaire, (score 1–6) median difference		0.30		<0.026	0.10–0.60	Grossarth 2007d [50]
												
		None (75)										

Cervix	IB-IVA	Iscador (102)				Self-regulation questionnaire, (score 1–6) median difference		0.25		<0.0005	0.15–0.35	Grossarth 2007f [51]
												
		None (102)										

Uterus	IA-C	Iscador (103)				Self-regulation questionnaire, (score 1–6) median difference		0.65		<0.0005	0.4–0.95	Grossarth 2008d [49]
												
		None (103)										

**Retrolective pharmaco-epidemiological cohort study**												

Breast	I–III	Conventional therapy, Helixor (167)				Odds ratio for occurrence of disease- or treatment associated symptoms: 0.508					0.319–0.811	Beuth 2008 [69]
												
		Conventional therapy (514)										

	I–III	Conventional therapy, Iscador (710)	Adverse drug reactions ↓, Odds ratio: 0.47		95% CI 0.32–0.67	Odds ratio for being symptom-free 3.56 (vomiting, headache, exhaustion, depression, concentration, sleep, dizziness, irritability) ↑					2.03–6.27	Bock 2004 [70]
												
		Conventional therapy (732)										

	I–IV	Conventional therapy, Eurixor (219)				Symptom mean score improved (nausea, appetite, stomach pain, tiredness, depression, concentration, irritability, sleep)				<0.0001		Schumacher 2003 [71,72]
												
		Conventional therapy (470)										

^I ^Chemotherapy (referring to the study by Piao et al.) – breast cancer: CAP, CAF (CAP: Cyclophosphamide, doxorubicin, cisplatin; CAF: Cyclophosphamide, doxorubicin, 5-fluorouracil); ovarian cancer: CP, IcP (CP: Cyclophosphamide, cisplatin, IcP: Ifosfamid, carboplatin); non-small cell-lung cancer: VP, MViP (VP: Vinorelbine, cisplatin; MViP: Mitomycin, vindesine, cisplatin).

^II ^Statistical significance of pre-post difference within each group

QoL: Quality of life; KPS: Karnofsky Performance Status Scale SCE: Sister chromatid exchange; ↑: increase; ↓: decrease. P-value, 95% CI: Statistical significance of difference between mistletoe (or other verum) and control group; n.s.: not statistically significant; EC: Epirubicin, cyclophosphamide (F: 5-fluorouracil); VEC: Vindesine, epirubicin, cyclophosphamide; CMF: Cyclophosphamide, methotrexate 5-fluorouracil; CAF: Cyclophosphamide, doxorubicin, 5-fluorouracil.

**Table 6:** Single-Arm Cohort Studies (e.g. Phase II Trials) on VAE Treatment in Breast and Gynaecological Cancer

**Author, Year**	**Treatment**^I^					**Site**^II^	**Outcome**^III^					**N**^IV^	**Quality Criteria Fulfilled**^VI^					
						
	**Preparation**	**Injection site**	**Dosage**	**Escalating dosage**	**Duration**		**CR**	**PR**	**NC**	**PD**	**QoL**		**L**	**M**	**N**	**O**	**P**	**Q**
**Breast, Ovary, CIN**																		

Mansky 2008 [44,73]	Helixor (& gemcitabine)	sc	Up to 250 mg, daily	Yes	9 w	Breast, others	0%	10%	47%	43%		27	(+)	+	+	-^V^	(+)	(+)

Schink 2006 [45]	Helixor (& surgery)	sc	3/week, varying individually	Yes	Up to 2 years	Breast, colon	-	-	-	-	↗^IIIa^	40	+	+	(+)	(+)^V^	(+)	-

Schöffski 2004 [32]	Aviscumine	iv	10 – 6400 ng/kg, 2/w	Yes	3–24 w, median 6 w	Ovary, breast, others	0%	0%	30%	70%		37	+	(+)	+	+	+	+

Mahfouz 1999 [74]	Viscum fraxini	sc or it	1 × 45 mg/w	No	16–136 w	Breast	8%	54%	35%	4%	↗	26	(+)	(+)	+	(+)	+	+

Mahfouz 1998 [75]	Abnobaviscum Fr	sc	1 × 45 mg/w	No	17 w	Breast	0%	44%	33%	22%	↗	9	-	(-)	(+)	-	-	(+)

Finelli 1998 [76]	Lektinol	sc	2,5 μl/kg, 2/w	No	Up to 12 w	Breast, others	-	-	-	-	↗	884	+	+	+	-	+	+

Portalupi 1995 [77]	Iscador M	sc	2 × 1 ng MLI/kg bw × w	No	16 w	CIN I–III	41%	27%	27%	5%		22	+	+	+	+	+	(+)

**Malignant effusion**																		

Bar-Sela 2006 [46]	Iscador M	ip	10 mg	No	repeatedly	Ascites (ovary, others)	Increase of interval between two successive paracenteses from 7 to 12 days, p = 0.001^IIIb^				↗^IIIc^	23	(+)	(+)	+	(+)	+	+

Werner 1999 [78]	Abnobaviscum Fr	ipl	1 × 75 mg/w	No	3–8 w	Pleural effusion (breast, others)	88%				↗	32	+	+	+	-	(+)	(+)

Stumpf 1994 [79]	Helixor A, M or P	ipl	100–1000 mg	Yes	repeatedly	Pleural effusion (breast, others)	61%	11%	22%			18	+	+	+	(+)	+	+

Friedrichson 1995 [80]	Helixor A, M	ip	100–1000 mg, 2/w	Yes	repeatedly	Ascites (ovary, others)	70%				↗	12	(+)	(-)	+	-	(-)	+

^I ^sc: subcutaneous, it: intratumoural, ipl: intrapleural, ip: intraperitoneal; iv: intravenous infusion; bw; body weight; w: week

^II ^CIN: cervical intraepithelial neoplasia. Stage: advanced, except in Portalupi 1995, and partly Schink 2006 and Finelly 1998; plural effusion and ascites indicates treatment site

^III ^CR: complete, PR: partial remission, NC: no change, PD: progredient disease, QoL: quality of life, ↗: improved, ↘ impaired

^IIIa ^Especially physical functioning, role, fatigue, appetite

^IIIb ^Median values, comparable abdominal circumference and symptom score or drained fluid before or during each paracentesis respectively

^IIIc^Trend improvement in symptom score, especially abdominal pain, abdominal pressure, and waking up at night due to shortness of breath

^IV ^N: Number of participants

^V ^Concomitant conventional oncological cytoreductive therapies in some of the patients

^VI ^L Well-described patient characteristic and disease (diagnosis, stage, duration), prognostic factors

M Outcome parameter relevant and well described

N Well-described intervention

O Concomitant therapies well described

P Outcome clearly described, temporal relationship between applied therapy and observed outcome precisely described

Q Selection of patients excluded

+ = adequately fulfilled, (+) = partly fulfilled, (-) = little fulfilled, - = not fulfilled

### Controlled studies

The 19 RCTs [47-63] (Table 1) encompassed 2420 participants, 16 non-RCTs [49-53,59,64-72] (Table 2) encompassed over 6399 participants (the sample size of one control group was not published). Cancer sites studied were breast (n = 20), uterus (n = 4), ovary (n = 6), cervix (n = 4), and genital (n = 1). One RCT investigated malignant pleural infusion. 4 studies not only investigated gynaecological or breast cancer but other cancer types as well.

Stages ranged from early-detected to advanced disease. 33 studies had two arms, one trial had three, and one four arms. Endpoints were: survival (22 studies), tumour remission, recurrence or time to recurrence or metastases (8 studies), pleurodesis (1 study), QoL or coping with disease (11 studies), QoL or tolerability of concomitant chemotherapy, radiotherapy or surgery (13 studies). Length of follow-up varied from three days in one trial to – usually – months or years.

All treatment groups received conventional care when indicated, and most patients had undergone prior surgery. In 16 studies (9 RCTs and 7 non-RCTs) the combination of VAE treatment and concurrent chemotherapy, radiotherapy or surgery was investigated. 13 of these studies assessed reduction of side effects from these cytoreductive therapies. Three trials directly compared VAE treatment versus chemotherapy treatment or versus radiation and hormones [60,62,66]. In most studies VAE therapy was used at least partly in an adjuvant setting after surgery or radiotherapy.

The commercial VAE applied were Iscador^®^, Helixor^®^, Eurixor^® ^or Lektinol^®^. VAE dosage mostly followed general recommendations, starting with low doses and increasing to an individually still well-tolerated dosage, or treating according to lectin-content (in 6 trials) or leaving treatment modalities to the physician's discretion, which, it can likewise be assumed, followed general recommendations. VAE was injected subcutaneously except in three trials employing intravenous infusion or intrapleural instillation [48,60,65]. Treatment duration was often not specified and depended on primary endpoint and related follow-up, ranging from one single application (in one trial [65]) to repeated applications over months and years. Control groups either received no further comparison treatment (n = 27), additional placebo application (n = 5), doxycycline (n = 1), Lentinan (n = 1) or radiation and hormones (n = 1). 4 trials had double-blinded treatment application.

### Single-arm studies

11 prospective cohort studies [32,44-46,73-80] (Table 6) included 1,130 patients. Cancer sites studied were breast (n = 6), ovary (n = 1), CIN (n = 1), malignant pleural effusion (n = 2) and malignant ascites (n = 2). 8 studies investigated several cancer types. Tumour stages were advanced or inoperable except in three studies. In most studies patients had received conventional treatment some time previously. Directly preceding or concurrent anti-cancer treatment had been applied in two studies (gemcitabine [44], surgery [45]). Nine studies assessed tumour remission; seven reported QoL or symptomatic relief. Two studies primarily investigated the toxicity profile, pharmakokinetics and potential interactions of either the combination of gemcitabine and VAE [44,73] or of rML [32], and secondarily assessed tumour behaviour. The commercial VAE remedies were Abnobaviscum^®^/Viscum fraxini, Iscador, Helixor, Lektinol or Aviscumine^® ^(rML). VAE were applied subcutaneously (n = 6), intratumourally (n = 1), intrapleurally (n = 2), intraperitoneally (n = 2) or as an intravenous infusion (n = 1). Dosage depended on the preparation and mode of application; some treated according to lectin content, others started with a low dosage and increased successively, or started with high dosage and applied it consistently once weekly. For intrapleural and intraperitoneal (repeated) application, VAE was diluted in 5 to 15 ml or 100 ml solution. Treatment duration and follow-up ranged from weeks to, most commonly, months or years.

### Quality assessment

Table 1, 2 and 6 summarize the validity assessment. Methodological quality differed substantially in the reviewed studies. 19 trials had randomized treatment allocation. The RCTs were mostly small (median sample size n = 60, range 23–692), particularly when investigating survival (median n = 52). Although RCTs investigating QoL were only slightly larger (median n = 68), they nevertheless encompass 4 trials that largely met modern standards of clinical trials and three of them had a sample size above 200. In four of the RCTs the patients and physicians were blinded; three further RCTs had an active or a placebo control-treatment. – 16 studies were non-randomized (median sample size n = 203, range 82–1442), 15 of them had controlled for confounding by close prospective (in one case retrospective) pair matching, by alternating treatment allocation and by multivariate analysis or propensity score (though in one study only for the main outcome parameter [69]). – Assurance of data quality according to ICH-GCP ("Good Clinical Practice") or GEP ("Good Epidemiological Practice") guidelines was reported in 5 RCTs and 4 non-RCTs. Eight of the RCTs and 8 of the non-RCTs were embedded in the same large epidemiological cohort study. Most studies did not present a clear documentation of co-interventions. Regarding the other quality aspects, most studies – especially the more recent ones – were reasonably well designed and conducted.

In the single-armed studies, study quality was reasonably good except in an unpublished report [80] and in an abstract publication [75] with too little information. Two studies had applied VAE in combination with or subsequent to conventional cancer treatment and one study had explored CIN, which has high spontaneous remission rates.

### Characteristics of the preclinical studies

The *in vitro *cytotoxicity of different VAEs as well as isolated or recombinant lectins or their A-chain, viscotoxins, or other protein fractions were tested with different methods in a variety of human breast, ovarian, uterine, vulvar and cervical cancer cells [12,20,22,81-110] (Table 7).

**Table 7:** *In-vitro *Studies on Cytotoxicity of VAE in Human Breast or Gynecological Cancer Cells

**Tumour cell**	**VAE**	**Result**		**Reference**
**Breast cancer**				

MFM-223	Iscador Qu, M, A Iscador P ML I	IC_50_	0.05–0.12 mg/ml 1.89 mg/ml 38 ng/ml	[22]
	
	Iscador M, Qu, Abnobaviscum Fr	Inhibition of proliferation	0.1–1 mg/ml 0.01–1 mg/ml	[81]

KPL-1	Iscador Qu, M, A Iscador P ML I	IC_50_	0.1–0.3 mg/ml 1.94 mg/ml 141 ng/ml	[22]
	
	Iscador M, Qu, Abnobaviscum Fr	Inhibition of proliferation	1 mg/ml 0,1–1 mg/ml	[81]
	
	Iscucin^® ^A, M, P, C, Po, T, Qu, S	Cytotoxicity	0.1 mg/ml	[82]
	
	Iscador M ML I	No stimulation of cell proliferation	0.05–5 ng ML/ml 0.01–5 ng/ml	[83]

MCF-7	Iscador Qu, M, A Iscador P ML I	IC_50_	0.09–0.12 mg/ml 1.61 mg/ml 410 ng/ml	[22]
	
	Lektinol	IC_50_	>10 ng ML I/ml	[84]
	
	Iscador Qu, M, P (max. 1 or 1.5 mg/ml)	Inhibition of S-phase progression Induction of apoptosis		[85-87]
	
	Iscador M Iscador P ML I Iscador Qu	IC_50_ No influence	185 μg/ml no activity 0.003 μg/ml 0.0015–15 μg/ml	[88,89]
	
	Viscotoxin isoforms (A1, A2, A3, B, 1-PS) Viscotoxin isoform U-PS	GI_50_ LC_50_	0.02–0.8 μg/ml 0.6 to >1 μg/ml no activity	[90]
	
	ML I A chain	Inhibition of proliferation	0.5 μg/ml	[91]
	
	ML I, ML II, ML III	Inhibition of proliferation	1–10 ng/ml	[91]
	
	TNF & ML I (100 ng/ml)	Potentiation of TNF-cytotoxicity		[92]
	
	Lektinol	IC_50_	0.003 μg/ml	[93]
	
	Helixor P ML I	IC_50_	> 150 μg/ml 0.086 μg/ml	[94]
	
	Iscucin M, P, C, Po, T, Qu, S Iscucin A, Pi	Cytotoxicity	0.1 mg/ml no activity	[82]

MCF-7/ADR	Lektinol	IC_50 _(SRB assay)	0.3 E-4 μg/ml	[93]

MAXF 401NL	Helixor P ML I	IC_50_	0.66 μg/ml 0.003 μg/ml	[94]
	
	Iscador M Iscador P ML I Iscador Qu	IC_50_ >70% growth inhibition	< 3 μg/ml no activity 0.353 E-4 μg/ml 10 μg/ml	[88,89]

MAXF 401	Lektinol	IC_50_	< 0.1 E-4 μg/ml	[93]

MAXF 1162	Lektinol	IC_50_	< 0.1 E-4 μg/ml	[93]

MAXF 449	Lektinol	IC_50_	0.2 E-4 μg/ml	[93]

MAXF MX1	Lektinol	IC_50_	< 0.1 E-4 μg/ml	[93]

MDA-MB-231	Lektinol	IC_50_	0.7 E-4 μg/ml	[93]
	
	Helixor P ML I	IC_50_	135 μg/ml 0.041 μg/ml	[94]

MDA-MB-468	Helixor P ML 1	IC_50_	47 μg/ml 0.006 μg/ml	[94]

MDA-MB-486-HER2	Iscador M	Inhibition of epidermal growth factor-induced proliferation	0.5 μg/ml	[95]

Colo-824	Iscador M ML I	No stimulation of cell proliferation	0.05–5 ng ML/ml 0.01–5 ng/ml	[83]

HCC-1937	Iscador Qu, M, A Iscador P ML I	IC_50_	0.1 to 0.3 mg/ml 2.14 mg/ml 320 ng/ml	[22]
	
	Iscucin A, M, P, C, Po, T, Qu, S	Cytotoxicity	0.1 mg/ml	[82]

BT474	Helixor M, A	Cytotoxicity (WST-1)	Maximum (80 and 100%) with 25 mg/ml	[96]

Primary breast cancer	Iscador M, Qu Abnobaviscum Fr	Mitochondrial activity (MTT)	50–80% with 0.1–0.001 mg/ml	[81]
	
	Abnobaviscum M	Inhibition of proliferation	0.5–50 μg/ml	[97]
	
	ML I	Inhibition of proliferation	1–50 ng/ml	[20,98]

T47D	ML I, II, III	IC_50_	> 0.1 – 1 ng/ml	[99]
	
	ML I A-chain	Inhibition of proliferation	10 ng/ml	[91]

BT549	ML I A-chain	Inhibition of proliferation	500 ng/ml	[91]

HBL100	ML I A-chain	Inhibition of proliferation	100 ng/ml	[91]

Breast cancer cells	ML II, ML III, viscotoxins	Cytotoxicity		[100]

**Ovarian cancer**				

OVXF 1619L	Helixor P ML I	IC_50_	119 μg/ml 0.100 E-3 μg/ml	[94]

OVXF 899L	Helixor P ML I	IC_50_	>150 μg/ml 0.229 μg/ml	[94]

SKOV-3 (HER-2 expression)	Recombinant ML I	IC_50_ Induction of apoptosis	0.033 ng/ml	[101]

OVCAR3	Iscador Qu, M (max. 1 or 1.5 mg/ml)	Inhibition of S-phase progression, Induction of apoptosis	No clear effect	[87]

OVXF 899	Lektinol	IC_50_	0.3 E-3 μg/ml	[93]

OVXF 1353	Lektinol	IC_50_	0.01 μg/ml	[93]

OVXF 1023	Lektinol	IC_50_	< 0.1 E-4 μg/ml	[93]

SKOV3	Lektinol	IC_50_	< 0.1 E-4 μg/ml	[93]

Primary ovarian cancer	Abnobaviscum M	Inhibition of proliferation	5 μg/ml	[97]

**Uterine cancer**				

UXF 1138L	Iscador M Iscador P ML I Iscador Qu	IC_50_ Growth inhibition >30%	6.8 μg/ml No activity 0.16 E-4 μg/ml 15 μg/ml	[88,89]

UCL SK-UT-1B	Helixor P ML I	IC_50_	> 150 μg/ml 0.038 μg/ml	[94]

SK-UT-1B	Lektinol	IC_50_	0.6–5.5 ng ML I/ml	[84]
	
	ML I	Inhibition of proliferation	0.5–500 ng/ml	[98,102]
	
	Iscador M ML I	No stimulation of cell proliferation	0.05–5 ng ML/ml 0.01–5 ng/ml	[83]

SK-UT-1	ML I	Inhibition of proliferation	0.5–500 ng/ml	[98,102]

MES-SA	ML I	Inhibition of proliferation	0.5–500 ng/ml	[98,102]

Primary uterus cancer	Abnobaviscum M	Inhibition of proliferation	5–50 μg/ml	[97]

**Vulvar cancer**				

SK-MLS-1	Lektinol	IC_50_	2 to >5 ng ML I/ml	[84]
	
	ML I	Inhibition of proliferation:	0.5–500 ng/ml	[98,102]
	
	Iscador M ML I	No stimulation of cell proliferation	0.05–5 ng ML/ml 0.01–5 ng/ml	[83]

**Cervical cancer**				

	HeLa	TNF & ML I (100 ng/ml)	Potentiation of TNF-cytotoxicity	[92]
	
	ML I	Inhibition of protein synthesis	100 μg/ml	[12,103]
	
	Protein fractions	Complete inhibition of DNA-, RNA-synthesis Proliferation	1 μg/ml no effect	[104]
	
	Viscotoxins	IC_50_	0.2–1.7 μg/ml	[105]
	
	Helixor M	Growth inhibition	≥ 0.01 mg/ml	[106]
	
	Isorel^®^	Cytotoxicity	30 μg/μl	[107]
	
	Isorel A, M, P, ML I	Cytotoxicity	> 1 μl/ml > 1 μg/ml	[108]
	
	Iscador M Helixor M VAE M	LC_50_	16 μg/ml 35,4 μg/ml 3,9 μg/ml	[109,110]
	
	Iscador M, Qu Abnobaviscum Fr	Growth inhibition	0.1–1 mg/ml 0.01 mg/ml	[81]

GI_50_: 50% growth inhibitory concentration

LC_50_: 50% lethal concentration

IC_50_: 50% inhibitory concentration

MCF-7/ADR: adriamycin(doxorubicin)-resistant MCF-7 cell line

HER: human epidermal growth factor receptor

#### Animal studies

43 studies were found. 9 of these were excluded as they investigated: tumour-bearing eggs [111], pre-incubation of tumour cells with VAE [112,113], different cancer types without differentiating the results accordingly [114], or isolated VAE proteins that were unstable [115]. Of the remaining 34 experiments [96,111,116-134] (Tables 8 and 9), 28 had been conducted in mice and 6 in rats. 22 experiments had included 788 animals, (5–20 per treatment group), one included 282 VAE-treated animals (number of control animals were not reported), the other reports gave no details. 32 experiments investigated breast tumours (15 of these Ehrlich carcinoma, ECa), one uterus epithelioma and one ovarian cancer. 28 had used murine tumour models, 5 were of human origin and 1 an autochthonous model (methylnitrosurea-induced tumourigenesis). 24 experiments investigated whole VAE (two of these VAE-activated macrophages), two investigated isolated MLs, two rMLs, two investigated other isolated proteins, and four investigated polysaccharides ("Viscumsäure"). VAE were applied systemically in 17 experiments (subcutaneous, intraperitoneal, intratumoural on opposite site, intramuscular), local at the tumour site in 15 experiments (intraperitoneal, intratumoural, intramuscular), and without specification in two studies.

**Table 8:** Animal Studies of VAE on Breast or Gynaecological Cancer (transplanted human or murine tumours or primary autochthonous tumour)

**Tumour, site**	**Animal**	**VAE, application and dosage**	**Tumour growth** **T/C**	**Survival** **ILS**	**Other outcomes**	**Reference**
**Human breast**	**Mice**					

MAXF 449, sc	Nude mice	Local Abnobaviscum Qu 8 or 4 or 2 mg/kg, it, qd * 3	6 to 20%			[116]
					
		Systemic Abnobaviscum Qu 8 mg/kg, it, qd * 3	78%			

MAXF 449, sc	Nude mice	Abnobaviscum M 8 mg/kg, sc, qd * 3 * 2 w	68%			[116]

BT474, sc	Mice (BALB/c)	Helixor M or A 5 mg, it, qd * 3 * 2 w	29 to 52%			[96]

**Murine breast**						

Carcinoma, sc, iv	Mice (CBA/HZgr)	Isorel M, 3 mg, sc, qod * 21	No difference		Lung-metastases: VAE vs. control: 13.4 vs. 37.5	[117]

Carcinoma, sc	Mice (CBA/HZgr)	Isorel M, 1400 mg/kg, 2 w	20%			[118]

Carcinoma, sc	Mice (CBA/HZgr)	Isorel M, 140 mg/kg			Recurrence after resection, VAE vs. control: 47% vs. 78%	[118]

Carcinoma, iv	Mice (CBA/HZgr)	Isorel M, 140 mg/kg, ip			52 lung-metastases	[118]
						
		Endoxan, 50 mg/kg			23 lung-metastases	
						
		Isorel M, 140 mg/kg & Endoxan 50 mg/kg			10 lung-metastases	
						
		Control			76 lung-metastases	

C3H adenocarcinoma, 16/C	Mice (B6C3F1)	Iscador M, 50 or 100 mg/kg, ip, qd, day 1–14	28%	15 to 20%		[119]

RC adenocarcinoma, sc	Mice (DBA)	VAE ^I^, sc	20 to 40%			[111]

ECa, ip	Mice (NMRI)	VAE (supracritical CO_2 _extraction), 2 mL/kg, ip, qd, starting day -7, day 0, or day 7	65 to 100%^II^			[120]

ECa, ip	Mice (BALB/c)	Iscador, 15 μg, ip, day -1		108%		[121]
						
		Sodium caseinate & Iscador, 15 μg, ip, day -1		no death		
						
		Sodium caseinate, day -1		0%		

ECa, ip	Mice (BALB/c)	Iscador, 15 μg, ip, day 6		82%		[121]
						
		Sodium caseinate, day 6		7%		

ECa, ip	Mice (BALB/c)	Iscador-activated macrophages, ip, day 6		49%		[121]
						
		Non-activated macrophages, ip, day 6		4%		

ECa, ip	Mice (BALB/c)	Iscador activated macrophages, ip, day 6, 10, 14		98%		[121]
			
		Non-activated macrophages, ip, day 6, 10, 14		9%		

ECa, sc	Mice (BALB/c)	Iscador, 15 μg, it, day 7			Severe necrosis, infiltration of lymphocytes and macrophages	[122]

ECa, sc	Mice (Swiss)	Iscador M, 1.66 mg, im, qod * 5 or 10	3 to 10%			[123]

ECa, ip	Mice (Swiss)	Iscador M, 1.66 mg, ip, qod * 10		76%		[123]

ECa, ip	Mice (Swiss)	Iscador M, 25 or 50 mg/kg, ip, qd * 14		69 to 97%	No tumour-free mice	[119]

ECa, ip	Mice (Swiss)	Iscador M, sc, cumulative dose 4, 5, 150, or 200 mg		-4 to 0%		[124]

ECa, sc	Mice	VAE, it, 0.1–0.2 ccm, qod * 6–10			Complete remission & no recurrence: 27%	[125,126]

**Murine breast**	**Rats**					

Walker carcinosarcoma 256; sc	Rats (Sprague Dawley)	Iscador M, sc, cumulative dose 11, 16, 500, or 750 mg or combination of Iscador M, sc, cumulative dose 11 or 500 mg & Cetraria praeparata, cumulative dose 3 or 164 mg	93 to 115%	-16 to 8%		[124]

Dunning DMBA-5A; sc	Rats	Iscador M, 2.5–15 mg, ip, qd	No difference		Less tumour viability	[127]

Walker carcinosarkoma 256	Rats	Iscador M, 0.005–0.5 mg, im, qd	No difference		Metastases: no difference	[128]

**Autochthonous**						

Methylnitrosurea-induced	Rats (Sprague Dawley)	Iscador M c. Arg., sc, 0,2 ml/day, 50 mg/week * 6 weeks	75%	-16%		[124]

sc: subcutaneous; im: intramuscular; it: intratumoural; ip: intraperitoneal; iv: intravenous; w: week;

qod: every other day; qd: every day; T/C: treated tumour/control tumour; ILS: increase in life span

All experiments did have control groups, but these were only mentioned if necessary for results

^I ^Part of a screening programme for substances with anticancer activity (1,000 plant extracts from 107 plant species)

^II ^Relating to volume of ascites; effects greatest with therapy started on day -7

**Table 9:** Animal Studies of VAE Compounds in Breast or Gynaecological Cancer (transplanted human or murine tumours)

**Tumour, site**	**Animal**	**VAE**	**Tumour growth** **T/C (%)**	**Survival**	**Other outcomes**	**Reference**
**Human breast tumour**						

Breast	Mice	rML 0,3 ng/kg – 3 μg/kg, ip, qd * 5 * 2–4 w	No effect			[129]

**Murine breast tumour in mice**						

C3L5, adenocarcinoma; sc	Mice (C3H7HeJ)	ML I, 1 ng/kg, sc, q3d, day 7–19	160		27.6 lung-metastases	[130]
					
		IL-2, twice 6 × 104 IU/mouse, ip q8h 2 * qd * 5	43		2.3 lung-metastases	
					
		Combination of ML 1 & IL-2	37		2.3 lung-metastases	
					
		Control			7.5 lung-metastases	

ECa, ip	Mice (ICR)	ML I, 80 ng, ip, day 1		70% died after 50 days		[131]
						
		A-chain of ML I, 100 μg, ip, day 1		80% died after 57 days		
						
		B-chain of ML I, 10 μg, ip, day 1		80% died after 58 days		
						
		Control		100% died after 20 days		

ECa, sc	Mice (BALB/c)	VAE 5 kDa peptides, 2 μg, it, day 7			Severe necrosis, infiltration of lymphocytes and macrophages	[122]

ECa, ip	Mice (CD-1)	Vester' Proteins, ip, 0.1 or 1 or 10 μ/kg, qd * 10		ILS: 0, 33, and -33%I		[132]

ECa	Mice	Polysaccharide („Viscumsäure“), ip, qd * 6	Slight effect			[133]

Adenocarcinoma EO 771	Mice	Polysaccharide („Viscumsäure“), ip, qd * 6	Moderate effect			[133]

**Murine breast tumour in rats**						

Walker Carcinosarcoma	Rats	Polysaccharide („Viscumsäure“), ip, qd * 6	Moderate effect			[133]

**Other gynaecological tumour**						

Ovary, SoTü 3, ip	Mice (SCID)	rML 30 ng/kg, ip, qd * 5 * 12		35% mice alive at day 84	40% tumour-free mice at day 84	[134]
					
		rML 150 ng/kg, ip, qd * 5 * 12		10% mice alive at day 84	10% tumour-free mice at day 84	
					
		rML 500 ng/kg, ip, qd * 5 * 12		75% mice alive at day 84	65% tumour-free mice at day 84	
					
		Control		15 mice alive at day 84	10% tumour-free mice at day 84	

Uterusepithelioma T-8 Guérin	Rats	Polysaccharide ("Viscumsäure"), ip, qd * 6	Moderate effect			[133]

All experiments did have control groups, but these were only mentioned if necessary for results.

sc: subcutaneous; it: intratumoural; ip: intraperitoneal; iv: intravenous; w: week;

qod: every other day; qd: every day; T/C: treated tumour/control tumour; ILS: increase in life span.

^I ^Application of 10 μg/kg of proteins had toxic effects

These experiments had been conducted in Germany, Switzerland, Austria, USA, India, Croatia and Serbia. 9 of the 34 experiments reported the funding source, 8 of these had public funding and one a combination of public and industry funding. 19 had been published since 1990 and 15 before (1938–1989). 21 were published in peer-reviewed and 2 in other journals, 6 were published in scientific reference books, 1 as a conference abstract, and 4 in a patent specification. Published information was often insufficient and sometimes extremely sparse. 6 experiments reported randomized treatment allocation. Regarding the control group, placebo treatment was described in 13 experiments – five of these with identical application schedule to the verum treatment -, no treatment in 11 experiments, and 9 experiments gave no information. None of the experiments reported a blinded outcome assessment (but randomized treatment allocation and blinded outcome assessment are generally routine practice).

### Outcome

We found substantial heterogeneity of the studies in terms of intervention, patient characteristics, clinical diagnosis, measured outcomes, design, methodological quality and potential positive and negative biases. We therefore regarded quantification of effect size by combining results as unreliable and decided on a non-quantitative synthesis and discussion. A subgroup of studies (2 RCTs, 2 non-RCTs on breast cancer), with a comparable design (all originating in the same epidemiological cohort study) had already been analysed in a quantitative meta-analysis [135].

Results of controlled clinical studies are shown in Table 3 (survival), Table 4 (tumour behaviour) and Table 5 (QoL and tolerability of conventional cancer treatment); results of single-arm studies are shown in Table 6.

Results of the preclinical studies are presented in Tables 7, 8 and 9.

### Breast cancer

#### 

*Clinical studies: Survival *(Table 3) was investigated by 4 RCTs and 3 non-RCTs (one of these is shown with three subgroups in Table 3): Two RCTs reported a statistically significant benefit of VAE (of these one also included other tumour sites, and the other suffered from a major attrition rate without preventing bias by an intention-to-treat analysis), and two RCTs reported a small positive trend. The results of the latter two RCTs were also combined in an individual patient data meta-analysis; the result just missed significance (HR: 0.59, 95% CI: 0.34–1.02, p = 0.057) [135]. Two non-RCTs had observed a statistically significant benefit, and one a small positive trend. The results of two non-RCTs were additionally combined in an individual patient data meta-analysis, and showed highly significant results (HR: 0.43, 95% CI: 0.34–0.56, p < 0.0005) [135]. *Tumour behaviour *(Tables 4 and 6) was investigated by two RCTs, four non-RCTs and 4 single-arm studies. Four of the controlled studies combined VAE and conventional cancer treatment. These studies partly reported a benefit regarding disease recurrence and time to disease relapse and partly no difference; none found a disadvantage. Two single-arm studies reported tumour remission in 44–62% of patients after local application of high dosage VAE. Another study found no remission after the application of rML. *QoL *and the *reduction of side effects of chemotherapy, radiation and surgery *(Tables 5 and 6) were assessed by 11 RCTs, 6 non-RCTs and 4 single-arm studies: 19 of these 21 studies reported a benefit, mostly statistically significant, one study reported no QoL-benefit but a reduction of side effects, and the smallest of these studies found no difference. Three major pharmaco-epidemiological studies investigated patient charts and found reduced disease- and therapy-associated symptoms in VAE-treated groups.

In *preclinical studies *(Tables 7, 8, and 9) VAE and VAE compounds showed cytotoxic effects in cancer cells. VAE also counteracted growth factor-induced proliferation and migration in breast cancer cells [95]. In mice, VAE inhibited tumour growth in most cases, especially when applied locally and in high dosage. Survival was prolonged in most cases, and numbers of metastases and local recurrences were reduced after application of VAE or of VAE-activated macrophages; one study found no benefit. All experiments using local VAE application found a benefit in relation to survival and tumour-growth inhibition. In rats, no clear benefit of VAE could be seen. Results from applying isolated or recombinant VAE compounds were inconsistent: some moderate effects of proteins (e.g. lectins) or polysaccharides were observed in relation to survival and tumour growth, while others observed none or possibly also adverse outcomes.

### Cervical cancer

#### 

*Clinical studies: Survival *(Table 3) was investigated by one RCT and three non-RCTs: all four reported a beneficial outcome which, however, was statistically significant only in the non-RCTs. *Tumour behaviour *(Table 4) was investigated by one non-RCT, which could not find an effect on disease recurrence or metastases mainly because these events scarcely occurred. One single-arm study reported 41% complete and 27% partial remissions in CIN after VAE application. *QoL *(Table 5) was assessed in one RCT and one non-RCT; both reported a statistically significant benefit.

Regarding *preclinical studies *(Table 7), only HeLa cells were investigated; here VAE and protein fractions showed cytotoxic effects.

### Uterus cancer

#### 

*Clinical studies: Survival *(Table 3) was investigated by two RCTs and two non-RCTs; three reported a statistically significant benefit while one found no difference. *QoL *(Table 5) was assessed by one RCT and one non-RCT; both found a statistically highly significant benefit.

Regarding *preclinical studies *(Tables 7 and 9), VAE and isolated ML I showed cytotoxic effects in different human uterus cancer cells. Concerning animal experiments, a patent specification mentions "moderate" effects of mistletoe polysaccharides on tumour growth in uterusepithelioma.

### Ovarian cancer

#### 

*Clinical studies:* Two RCTs and two non-RCTs investigated the influence of VAE on *survival *(Table 3) and reported a benefit, one of each with statistical significance. *Tumour behaviour *(Table 4) was investigated by two RCTs, each combining VAE and chemotherapy (plus radiotherapy in one study): these reported comparable outcomes. The influence of VAE on *QoL *and *tolerability of chemotherapy and radiation *(Table 5) was investigated by three RCTs and one non-RCT; all of them reported a statistically significant positive effect. In one trial using an aggressive chemotherapy protocol, higher dosages of Cisplatin and Holoxan could be given in the VAE group as the side effects were less intense [63]. One single-arm study applied recombinant lectins in ovarian cancer but found no remission.

Regarding *preclinical studies *(Tables 7 and 9), VAE showed cytotoxic effects in various ovarian cancer cells. In SCID mice, rMLs led to increased survival and to more tumour-free animals at the highest and lowest dosage, while no effect was observed at the medium dosage.

### Genital cancer

#### 

*Clinical studies:* One non-RCT (published in 1963) reported partly improved disease-specific *survival *(Table 3). Regarding *preclinical studies *(Table 7), VAE showed cytotoxic effects in vulvar cancer cells.

### Malignant effusion

#### 

*Clinical studies:* One RCT and four single-arm studies investigated treatment of malignant pleural effusion and ascites (originating from breast or ovarian cancer, among other cancer sites), and all reported substantial remission rates (Tables 4 and 6).

### Safety

Tolerability was generally good. One case of urticaria and angioedema [56] and one case of "generalized reaction" [69] were described. Otherwise no major side effects or toxicity were reported. Frequent minor, dose-dependent and spontaneously subsiding symptoms included reactions at the injection site (swelling, induration, erythema, pruritus, local pain) and mild flu-like symptoms or fever. In one study, local reactions intensified during concomitant chemotherapy [64]. A higher prevalence of depression was documented in the unadjusted data of a retrolective non-RCT [69] in VAE-treated patients; these patients also had a higher prevalence of other treatments such as hormones. After intrapleural instillation, VAE induced significantly fewer side effects than doxycycline [60]. No indication for an interaction of VAE and chemotherapy could be found (i.e. remission rate) and VAE had no influence on the plasma concentration of gemcitabine [44,73]. No toxicity was observed in animal studies, except after application of high doses of an isolated protein complex with unknown constituents [132].

## Discussion

A variety of clinical studies and experiments have investigated the potential therapeutic effects of VAE and its compounds in breast and gynecological cancer, and predominantly reported positive effects. Nevertheless they have to be interpreted with caution and within their context.

The strongest and most consistent results from VAE in clinical studies concern *QoL *and improved *tolerability of conventional treatment*. QoL questionnaires included mostly well established and validated QoL instruments and one on psychosomatic self-regulation. The latter is a 16 item QoL instrument that measures competence and autonomy, in terms of the ability to actively adapt to stressful life situations and to restore well-being. [136] This tool has so far been exclusively used in studies focusing on complementary cancer treatments. Improvement was seen especially in relation to self-regulation, fatigue, sleep, nausea/vomiting, appetite, diarrhoea, energy, ability to work, enjoyment of life, depression, anxiety, pain, and general physical, emotional, and functional well-being (for more details see Kienle GS, Kiene H: Influence of mistletoe treatment on quality of life in cancer patients. A systematic review of controlled clinical studies. Submitted). Regarding the side effects of conventional oncology treatments, reduced hematopoetic damage (i.e. leukopenia) and immuno-suppression was reported by some, but not by all studies. Similar, less chemotherapy-related events were observed in some but not in all studies. Validity of this evidence is quite good. 15 RCTs are available, four of them double-blinded (three of them showing a positive result) and one with an active control treatment. 5 RCTs reported following ICH-GCP guidelines and three of them comprised more than 200 patients each. Questions remain regarding observation or reporting bias, which is of major importance in relation to subjectively assessed outcomes such as QoL and subjective symptoms. Treatment should therefore be blinded; but blinded subcutaneous VAE application can easily be correctly identified by doctors and patients [55,137], due to its local reactions and mild flu-like symptoms. In the four blinded trials reviewed here, a considerable degree of unblinding was detected by asking patients and physicians in one study [55]; and can be presumed in two other of these trials where substantially more VAE-treated patients reported local reactions than control patients [54,57]. Other RCTs did not blind treatment application, as blinding is unreliable. Therefore questions will remain in "blinded" as well as in open trials even though in general cancer or non-cancer trials could not detect relevant improvements of QoL or disease symptoms due to suggestive administration of inert substances [138-140]. Nevertheless, the frequency, magnitude, duration and conditions of QoL or symptomatic improvement in the course of VAE treatment should be clarified in more detail. Especially relevant might be the further elucidation of possible effects on cancer-related fatigue (see also [141]), which is one of the most disabling conditions in cancer patients, with only few therapeutic options for influencing it effectively [142-144]. Regarding simple pre-post assessments of QoL in single-arm studies, it is probably unnecessary to state that they are generally not appropriate for judging influences on QoL, since it is affected by many factors.

Concerning *survival *(Table 3), some of the RCTs show a statistically significant benefit while others show a statistical trend or no difference. Most of the non-RCTs (which included larger patient numbers) show a major impact. The validity of the studies is limited because of their small sample size (median only 52 participants per RCT), and because 8 of the 9 RCTs were imbedded in the same (large) epidemiological cohort study. This study was started in the 1970s, before modern standards of data quality control (ICH-GCP, GEP) were established, and it therefore does not fulfil modern standards in this respect. The 9^th ^RCT had enrolled more patients but was conducted even earlier, and suffers from a major attrition rate due to protocol violation [62]; the subsequent analysis followed the "as treated" instead of the "intention-to-treat" principle [145]. Hence bias cannot be excluded. None of the survival studies was blinded, but survival is generally not easily affected by observer bias or suggestive effects [138-140]. Seen altogether, although results were consistent, questions regarding survival remain and validity of evidence is moderate at best. An independent, GCP-conform trial with sufficient power would be desirable to further evaluate potential survival benefit.

Regarding *tumour behaviour*, evidence from RCTs is scanty; most benefits were shown in non-randomized studies. In single-arm studies of patients with no concomitant conventional cancer treatment, high-dose or local application of whole VAE led to substantial remission of tumour or malignant effusion. This was also observed in animal studies: local application resulted in tumour-growth inhibition and increased survival. However, this application and dosage is not standard and cannot be recommended widely due to potential risks of high dose or local application. With ordinary VAE application, schedule and dosage, spectacular tumour remissions tend to be the exception [20,36]. No tumour remission was observed after application of rMLs. Remission in CIN cannot be distinguished from spontaneous remission rates, which are frequent in this indication.

Apart from the discussed issues, the following validity aspects have to be considered: An attrition rate above 10% was present in 10 RCTs. In 5 of these RCTs [49-51,53], patients were excluded before baseline assessment. Here the patients were provisionally enrolled into the matching and pairwise randomization procedure; subsequently they were asked for informed consent, and were excluded from the study if they declined, together with their matched twin. Even though the risk of bias with this procedure is small, as the complete randomization unit (patient pair) is excluded, the preferred conservative quality assessment in this review assessed these studies as not having excluded a drop-out bias. Of the remaining 5 trials, one had protocol violations in about 20% of patients as discussed above [62], and one trial used an aggressive chemotherapy that inevitably had to be halted in several patients [63]. Three trials did not report details.

To reduce publication bias we also included unpublished studies and conducted a thorough literature search with extensive expert consultations. One unpublished RCT (Lektinol in breast cancer by Schwiersch et al.) could not be included as it was not released by the manufacturer. Beyond this, we cannot rule out the existence of unpublished and unknown RCTs, but we presume that no well-conducted, large-size and valid trials escaped our attention. – Regarding preclinical studies achieving completeness is nearly impossible. These experiments are usually explorative, for instance when plant extracts are chemically analysed for active compounds or for cytotoxic effects; in general only relevant results are published, but not results of non-relevant or non-working models or unstable chemicals. (Even in the reviewed experiments, often not all but only the noteworthy results were presented in detail.)

Regarding funding, 27 of 28 controlled studies published since 2000 reported their funding source: 11 studies received funding from the pharmaceutical industry alone, 16 studies (all by Grossarth et al.) had both industry and public funding. There was no difference of results depending on funding source.

Regarding non-RCTs, bias by self-selecting the treatment is usually present in raw data. In particular, patients who choose complementary treatments differ substantially from patients not choosing them [70,146]. It is therefore indispensable to conduct careful adjustment of baseline imbalances or matching [147-149]. This has been done to a varying degree in most studies except in one without any adjustment [64], and in another which only adjusted for the main outcome parameter but not for the other reported results [69]. Without any adjustment, no conclusions can be drawn regarding the applied treatment. When conducted and analysed carefully, non-RCTs can provide valuable information regarding external validity and effectiveness, as they can investigate treatment effectiveness under routine conditions without distortion by the artificial and selective conditions of an RCT's experimental situation [150].

In *preclinical studies*, VAE show substantial cytotoxic effects in cells originating from breast and gynaecological cancer, and display tumour-growth inhibition in animal studies. Cytotoxicity, especially of the MLs (which bind on human breast cancer cells [151]), may be the cause of tumour reduction after local, intratumoural application of VAE. If systemically applied, the cytotoxicity of the MLs is of less relevance, as it is inhibited by serum glycoproteins [152] and by anti-ML antibodies [153] which are produced after a few weeks of VAE application. Therapeutic effects of the MLs were inconsistent and not very impressive in the reviewed experiments. However, in other tumour types, MLs have also shown substantial growth-inhibiting effects (e.g. [154-157]). Interestingly, in two experiments, the application of VAE-activated macrophages in mice not directly treated with VAE also showed tumour-growth inhibiting effects, while the application of non-activated macrophages had no effects [121]. Similarly in melanoma, the application of VAE-activated splenocytes inhibited metastasis [158,159].

In general, the predictive reliability of the preclinical studies for clinical application is fairly limited in most instances. Clinical cancer disease is insufficiently mimicked by animal models, with major differences regarding age, general condition, co-morbidity, invasiveness, metastases, antigenicity, immune system etc. The results of preclinical screening, especially for treatment of solid tumours, have therefore been largely disappointing. The models currently regarded as best for cytotoxic substances use patient-derived tumours that grow subcutaneously or orthotopically in nude mice, as in several cases reviewed here. Immuno-active substances may however still be insufficiently assessed in immune-deficient animals, as the main components of the immune system are missing (nude mice, for instance, cannot generate mature T-lymphocytes). Nevertheless, these preclinical experiments can provide important additional information for detecting the possible anti-cancer effects of medicinal plants, their active compounds, their mode of action and potential risks [20,160-162].

### Safety aspects

Mistletoe therapy was well tolerated in the reviewed studies. Mild flu-like symptoms and local reactions at the injections sites are frequent, dose-dependent and self-limited. Allergic reactions can occur, and a few case reports of anaphylactic reactions exist [163-166]. A phase I study, conducted at the NCCAM/NCI, investigated safety, toxicity and drug interactions between VAE and gemcitabine [73] and reported good tolerability, with neither dose-limiting toxicity of the VAE nor any effects on the plasma concentration of gemcitabine [44]. Combination of VAE with chemotherapy or radiotherapy did not negatively influence remission rate in clinical and in animal studies [56,63,118]. A higher prevalence of depression in VAE-treated patients in one study was observed in raw data of a self-selected population, without adjustment of baseline imbalances. This difference can be ascribed to variations in the patient population; for instance, they differed markedly in the prevalence of hormone treatment. No toxicity was observed in animal experiments.

## Conclusion

Preclinical and clinical studies investigating the influence of VAE and its isolated compounds on breast or gynaecological cancer suggest a benefit, with the strongest evidence in relation to QoL and tolerability of conventional anti-cancer treatments. Regarding survival, evidence is less conclusive; most of the clinical studies had a very small sample size (RCTs) and were embedded in the same large cohort study; therefore an independent trial would be needed. Tumour-growth inhibition has been insufficiently assessed in prospective clinical trials. Tumour regression seems not to have been connected with regular low-dose subcutaneous VAE treatment, but with high dose and local application. The latter has not yet been thoroughly assessed and is not generally recommended.

## Abbreviations

AMED: Allied and Complementary Medicine; CI: confidence interval; CIN: cervical intraepithelial neoplasia; DNA: deoxyribonucleic acid; ECa: Ehrlich carcinoma; EORTC QLQ-C30: European Organization for Research and Treatment of Cancer Quality of Life Questionnaire-Cancer; EORTC QLQ-BR23: European Organization for Research and Treatment of Cancer Quality of Life Breast Cancer Questionnaire; FACT-G: Functional Assessment of Cancer Therapy-General; FLIC: Functional Living Index – Cancer; GCP: Good Clinical Practice; GEP: Good Epidemiological Practice; GLQ-8: Global Life Quality; GM-CSF: granulocyte macrophage colony-stimulating factor; HeLa cells: immortal cell line from Henrietta Lacks; HR: hazard ratio; ICH: International Conference on Harmonisation; IFN-γ: interferon-gamma; IL: interleukin; kDa: kilodalton; KPS: Karnofsky performance status scale; MDR+: multidrug resistant; ML: mistletoe lectin; NCCAM: National Center for Complementary and Alternative Medicine; NCI: National Cancer Institute; NHS: National Health Service; NK: neutral killer (cell); NLM: National Library of Medicine; non-RCT: non-randomized controlled trial; QoL: quality of life; RCT: randomized controlled trial; rML: recombinant mistletoe lectin; SCE: sister chromatid exchange; SCID mice: Severe Combined Immunodeficiency mice; T-cells: lymphocytes matured in thymus; TCM: Traditional Chinese Medicine Index; TNF-α: tumor necrosis factor-alpha; TNM: tumor, node, metastasis; VAE: *Viscum album *extracts; VisalbCBA: *Viscum album *chitin-binding agglutinin.

## Competing interests

IFAEMM has received restricted research grants from Weleda, Abnoba and Helixor for other projects not connected to this review.

## Authors' contributions

The study protocol was written by GK and HK. Studies were read by GK, HK, AG. Study quality was assessed by GK and HK. Data were extracted by GK and checked by AG and HK. MS contributed substantially to data acquisition, analysis and interpretation of preclinical studies. GK wrote the paper which was critically revised and finally approved by HK, MS and AG.
